# Do early luteal serum progesterone levels predict the reproductive outcomes in IVF with oral dydrogesterone for luteal phase support?

**DOI:** 10.1371/journal.pone.0220450

**Published:** 2019-07-30

**Authors:** Antoine Netter, Julien Mancini, Christophe Buffat, Aubert Agostini, Jeanne Perrin, Blandine Courbiere

**Affiliations:** 1 Department of Gynecology, Obstetrics and Reproductive Medicine, AP-HM La Conception, Pôle femmes parents enfants, Marseille, France; 2 Aix-Marseille University, APHM, INSERM, IRD, SESSTIM, Public Health Department, La Timone Hospital, BIOSTIC, Marseille, France; 3 Laboratoire de Biochimie et de Biologie Moléculaire, Hôpital de la Conception, Marseille, France; 4 Institut Méditerranéen de Biodiversité et d'Écologie marine et continentale (IMBE), Aix Marseille University, CNRS, IRD, Avignon University, Marseille, France; University of Crete, GREECE

## Abstract

**Objective:**

We sought to determine whether the early luteal serum progesterone (P_4_) level predicts the success of IVF treatment with oral dydrogesterone for luteal support.

**Method:**

This retrospective monocentric cohort study included 242 women who underwent IVF treatment with fresh embryo transfer (ET) between July 2017 and June 2018. The population was unselected, and women were treated according to our unit’s usual stimulation protocols. For the luteal phase support (LPS), all women were supplemented with a 10 mg three-times-daily dose of oral dydrogesterone beginning on the day of oocyte pick-up (OPU). Blood sampling was performed on the day of ET (Day 2–3 after OPU) to determine the early luteal serum progesterone level.

**Results:**

ROC curve analysis allowed us to determine two thresholds for the prediction of live birth using the early P_4_ level. Women who had early luteal P_4_ levels greater than 252 nmol/l had a significantly higher live birth rate (27.1%) than women with early luteal P_4_ between 115 and 252 nmol/l (17.2%) and women with early luteal P_4_ below 115 nmol/l (6.0%; p = 0.011). After a multiple regression analysis, an early luteal P_4_ level greater than 252 nmol/l was still associated with a higher chance of a live birth than a P_4_ between 115 and 252 nmol/l (OR = 0.40 [0.18–0.91]; p = 0.028) or a P_4_ below 115 nmol/l (OR = 0.10 [0.01–0.52]; p = 0.006).

**Conclusions:**

Our study suggests a positive association between early P_4_ levels and reproductive outcomes in IVF using oral dydrogesterone for luteal support. The inconsistencies between our results and those of other studies suggest that extrapolation is impractical. Further larger prospective cohort studies should be conducted to determine reliable thresholds that could be used to personalize luteal phase support.

## Introduction

Over the past decades, increasing efforts have been made to identify best practices for controlled ovarian stimulation (COS) and embryo culture for In Vitro Fertilization (IVF) treatment [1]. In contrast, the luteal phase subsequent to the COS and oocyte retrieval have received less interest for a long time [2]. By modifying gene expression, progesterone guides the endometrial secretory transformation and therefore plays a fundamental role in implantation and early embryologic development [3,4]. After COS, the endogenous secretion of progesterone by the granulosa of the corpus luteum is insufficient [5]. The luteolytic effect of the GnRH analogs or GnRH antagonists used for the COS, inhibition of the hypothalamic-pituitary axis by the supra-physiological secretion of steroids, and aspiration of the granulosa cells during oocyte retrieval are the main hypotheses for this inadequate secretion [6]. Luteal phase support (LPS) compensates for this deficiency by supplementing with either progesterone or an hCG or GnRH agonist [7].

In recent years, clinical investigators have shown a renewed interest in the luteal phase subsequent to ART treatments. Evidence is accumulating regarding the existence of an optimal range of P_4_ levels on the day of embryo transfer (ET) in frozen embryo transfer cycles with hormone replacement therapy [8–10]. In 2018, Thomsen et al. published the results of a large prospective cohort study of 602 women who underwent IVF treatment and had serum P_4_ levels measured on the day of ET [11]. The authors determined that very low P_4_ levels (< 60 nmol/l) on the day of fresh ET (Day 2–3 after oocyte pick-up (OPU)) were associated with decreased chance of success of the IVF treatment. This result allows us to consider a possible value of early luteal P_4_ monitoring and luteal rescue by increasing the P_4_ supplementation. Thomsen et al. also reported that high P_4_ levels (> 400 nmol/l) were associated with a decreased chance of live birth after IVF treatment. If confirmed by other studies, these thresholds could encourage the cancellation of ET for women with high early luteal P_4_ levels.

However, concerns have been raised regarding the statistical analysis of the aforementioned study [12], and further studies are needed to assess the reproducibility of the suggested thresholds before they can be used in common practice, in particular with different COS and LPS protocols.

A recent large phase III RCT demonstrated the noninferiority of oral dydrogesterone compared with micronized vaginal progesterone for LPS in terms of pregnancy rates and tolerability [13]. Thus, dydrogesterone was recently approved for LPS in IVF [14]. Furthermore, given the assumed preference for the oral route over vaginal route, dydrogesterone is presumed to become the new standard for LPS in IVF treatment according to some authors [15]. The early luteal phase P_4_ levels have never been studied in the context of the use of dydrogesterone for LPS [16].

Throughout this retrospective data analysis, we aimed to determine if early luteal P_4_ levels (on the day of fresh ET) could allow us to predict the success of IVF on an unselected population treated with oral dydrogesterone for LPS.

## Materials and methods

### Study design

We conducted a monocentric retrospective cohort study. Data were retrospectively and anonymously collected. Before the study began, it was approved by the ethics committee of Aix Marseille University. All patients were informed of the anonymous and retrospective use of their data and could refuse to participate by simple notification.

### Study population

We enrolled couples who underwent IVF or ICSI treatments in our ART unit between July 2017 and June 2018 and who had early luteal phase serum P_4_ levels measured on the day of fresh ET (Day 2–3 after OPU). Women were eligible for inclusion regardless of the reason for the infertility or the number of previous unsuccessful IVF or ICSI cycles.

### Ovarian stimulation protocols

The choices of COS protocols and gonadotrophin doses were made in accordance with the standards of our ART unit based on age, BMI, ovarian reserve, outcomes of previous IVF or ICSI cycles, and other comorbidities such as endometriosis or polycystic ovary syndrome (PCOS). GnRH antagonist protocols were generally used as the first choice, especially for patients with an expected low ovarian response and for women with PCOS; in contrast, GnRH long agonist protocols were used for patients with endometriosis or as a second choice for patients with an inadequate response to the previous GnRH antagonist protocol. Short GnRH agonist protocols were used for low responders who had already experienced several failures after previous stimulations.

Patients who were treated in a long GnRH-agonist protocol were downregulated using a unique IM injection of 3 mg triptorelin (Decapeptyl, Ipsen Pharma, France) on the 22^nd^ day of the preceding cycle. Ovarian stimulation started after 14 days of downregulation after checking that the endometrial thickness was less than 4 mm. Final follicular maturation was induced by a single injection of 250 μg of choriogonadotropin alpha (Ovitrelle, Merck Serono Europe Limited, United Kingdom) when two or more leading follicles reached a mean diameter of 17 mm.

If the GnRH antagonist protocol was used, ovarian stimulation started on cycle Day 2. Daily GnRH antagonist cotreatment was added from cycle Day 6. Final maturation could be induced by a single injection of 250 μg of choriogonadotropin alpha (Ovitrelle, Merck Serono Europe Limited, United Kingdom) or by a dual trigger (250 μg of choriogonadotropin alpha plus 0.3 mg of Triptorelin) when two or more leading follicles reached a mean diameter of 17 mm.

Ovarian stimulation was performed using either r-FSH, r-FSH/r-LH or hMG. Dose adjustments were performed according to ovarian response, as monitored by transvaginal ultrasound during treatment. OPU was performed 36 h after the ovulation trigger. IVF or ICSI was performed according to normal clinical practice.

### Luteal phase support

All patients received a 10 mg three-times-daily dose of oral dydrogesterone (Duphaston, Mylan Medical, France) starting on the day of OPU [14,15]. We systematically checked with the patients’ compliance with this treatment on the day of ET. No additional treatment (neither hCG nor GnRH agonist bolus) was ever used during the luteal phase. Oral dydrogesterone administration continued from the day of OPU until the day of pregnancy testing or until the seventh gestational week (in the case of pregnancy).

### Hormone analysis

On day 2–3 after OPU, immediately before ET, blood samples were collected for P_4_ and estradiol determination. We used an automated Cobas e411 instrument (Roche Diagnostics, Mannheim, Germany) and the same assays for all hormone measurements during the entire study. Samples were tested by an electrochemiluminescence immunoassay for Progesterone III (Cobas 07092539 190). The intra- and inter-assay variation coefficients for the P determinations were 3.3% and 5.2%, respectively, and sensitivity was 0.2 ng/ml.

### Endpoints

hCG serum level was measured 14 days after ET and considered positive if hCG > 10 UI/ml. Clinical pregnancy was defined as the presence of a live fetus within an intrauterine gestational sac upon ultrasound examination at gestational weeks 6–7. Early pregnancy loss was defined by positive hCG testing at Day 14 and the absence of a live fetus within an intrauterine gestational sac upon ultrasound examination at gestational weeks 6–7. Thus, this definition includes patients with decreasing hCG after the first test, patients with no intrauterine gestational sac during the first ultrasound, patients with ectopic pregnancy and patients with a visible embryo without cardiac activity. Clinical pregnancy loss was defined as the loss of a viable intrauterine pregnancy between the first ultrasound up to and including gestational weeks 24 + 0. A live birth was defined as the delivery of a live infant after gestational week 24 + 0. Clinical gestational dating was performed using the day of OPU as gestational week 2 + 0 [11].

### Statistical analysis

Data are presented as percentages for categorical variables, as means and standard deviations for continuous parametric variables and as medians and ranges for continuous nonparametric variables.

First, a receiver operating characteristic (ROC) curve was used to evaluate the ability of early luteal P_4_ levels to predict live birth and to determine thresholds that could discriminate patients while maximizing sensitivity (Se) and specificity (Sp) with a good Youden index (*Se + Sp– 1*). Three P_4_ groups were thus formed using the optimal thresholds found (< 115 nmol/l, [115–252] nmol/l and > 252 nmol/l). ANOVA and χ^2^ tests were used, respectively, to compare continuous and categorical characteristics among the three P_4_ groups.

Second, the multiple logistic regression model used by Thomsen et al. was applied to assess the differences in terms of reproductive outcomes among the aforementioned P_4_ groups [11]. The model included the independent variables maternal age, maternal BMI, final follicle count on the day of trigger (>14 mm) and late follicular P_4_ level (>4.77 nmol/l) to estimate positive hCG levels, clinical pregnancy and live birth. For estimates of early pregnancy loss, adjustments were made for maternal age, maternal BMI, smoking, final follicle count and peak estradiol level on the day of trigger.

Third, to compare our results to those of the Thomsen et al. study, we conducted a multiple logistic regression by retaining only patients who responded to the same inclusion criteria (age < 41, BMI < 35, and excluding the flare up protocols). We compared the same P_4_ groups (< 60 nmol/l; [60–100 nmol/l]; [100–400 nmol/l]; > 400 nmol/l) to determine the differences in terms of reproductive outcomes.

All statistical analyses were two-tailed, and results were considered significant when p-values < 0.05 were obtained. These analyses were performed using IBM SPSS Statistics 20.0 (IBM Inc., New York, USA).

## Results

### Study population and reproductive outcomes

During the selected time period, 242 women who were undergoing IVF treatment at our ART unit had a blood sample on the day of ET and were included in the study. Baseline characteristics of participants are provided in Table 1, and cycle characteristics with crude reproductive outcomes are presented in Table 2. The mean age of patients enrolled was 34.9 ± 4.7 years, and the mean BMI was 24.8 ± 5.3 kg/m^2^. After IVF treatment, 69 (28.5%) patients had a positive hCG level and 22 (9.1%) patients had early pregnancy loss, leaving 47 (19.4%) patients with clinical pregnancy. Overall, 43 (17.8%) patients gave birth to live children.

**Table 1:** Baseline characteristics.

Progesterone (nmol/l)	N	All	< 115	115–252	> 252	P
Number of patients, n		242	50	122	70	
Maternal age (years)	242	34.9 ± 4.8	37.4 ± 3.9	34.5 ± 4.6	33.7 ± 5.0	<0.01**
Maternal BMI (kg/m^2^)	242	24.8 ± 5.3	27.5 ± 6.8	23.8 ± 4.15	24.5 ± 5.2	<0.01**
Maternal smoking (%)	242	21	20	25	14	0.235
Basal FSH (IU)	236	6.8 (1.7–16.3)	7.5 (3.6–13.9)	7.2 (1.7–16.3)	6.2 (2.4–13.4)	0.001**
Basal LH (IU)	234	5.0 (0.1–35.0)	4.5 (1.6–17.0)	5.1 (1.4–35.0)	4.9 (0.1–23.0)	0.549
Basal AMH (IU)	242	1.9 (0.1–17.0)	1.1 (0.1–11.5)	1.9 (0.1–17.0)	2.9 (0.5–9.8)	<0.01**
Antral follicle count, n	217	13 (2–55)	11 (2–55)	13 (4–38)	16 (4–42)	<0.01**
Primary diagnosis	242					
Unexplained (%)		23	16	33	10	<0.01**
Tubal (%)		27	32	25	27	0.608
Endometriosis (%)		13	14	14	11	0.871
PCO/PCOS (%)		12	8	7	23	<0.01**
Male (%)		25	30	21	29	0.377

Descriptive data are presented as the mean ± SD for continuous parametric data and as the median (range) for continuous nonparametric data. Categorical data are presented as percentages (%).

SI conversion factor for P4: nmol/l = 3.18 ng/ml.

**p< 0.01

**Table 2:** Descriptive data for controlled ovarian stimulation, oocytes, embryo transfer, and reproductive outcomes.

Progesterone (nmol/l)	N	All	< 115	115–252	> 252	P
Number of patients, n (%)		242	50 (21%)	122 (50%)	70 (29%)	
Protocol	242					
Antagonist (%)		64	60	60	76	0.066
Long GnRH agonist (%)		28	22	33	21	0.449
Flare up (%)		8	18	7	3	0.011*
Total FSH dose (IU)	242	2925 (1000–7200)	3450 (1125–6750)	2850 (1125–7200)	2475 (1000–6750)	<0.001**
Stim duration (days)	242	10.5 ± 1.7	10.3 ± 1.9	10.4 ± 1.7	10.7 ± 1.6	0.399
Final follicle count >14 mm on trigger day	240	8 (2–24)	5 (2–17)	8 (3–16)	10 (2–24)	<0.001**
Mode of triggering for final oocyte maturation	242					0.353
hCG (%)		69	62	70	75	
Dual trigger (%)		31	38	30	25	
Number of mature oocytes retrieved (n)	242	8 (1–25)	4 (1–14)	8 (1–20)	12 (2–25)	<0.001**
Number of fertilized oocytes (n)	242	5 (1–19)	3 (1–8)	5 (1–17)	6 (1–19)	<0.001**
Single embryo transfer (%)	242	26	40	24	21	0.048*
Double embryo transfer (%)	242	74	60	76	79	
At least one top quality embryo for transfer (%)	242	25	26	26	21	0.741
Positive hCG, n (%)	242	69 (28.5%)	9 (18.0%)	33 (27.0%)	27 (38.6%)	0.043*
Early pregnancy loss, n (%)	242	22/69 (31.9%)	5/9 (55.6%)	11/33 (33.3%)	6/27 (22.2%)	0.182
Clinical pregnancy, n (%)	242	47 (19.4%)	4 (8.0%)	22 (18,0%)	21 (30.0%)	0.009**
Live birth, n (%)	242	43 (17.8%)	3 (6.0%)	21 (17.2%)	19 (27.1%)	0.011*

Descriptive data are presented as the mean ± SD for continuous parametric data and as the median (range) for continuous nonparametric data. Categorical data are presented as percentages (%).

SI conversion factor for P4: nmol/l = 3.18 ng/ml.

*p<0.05

**p<0.01

### Threshold and P_4_ groups

**Fig 1:**
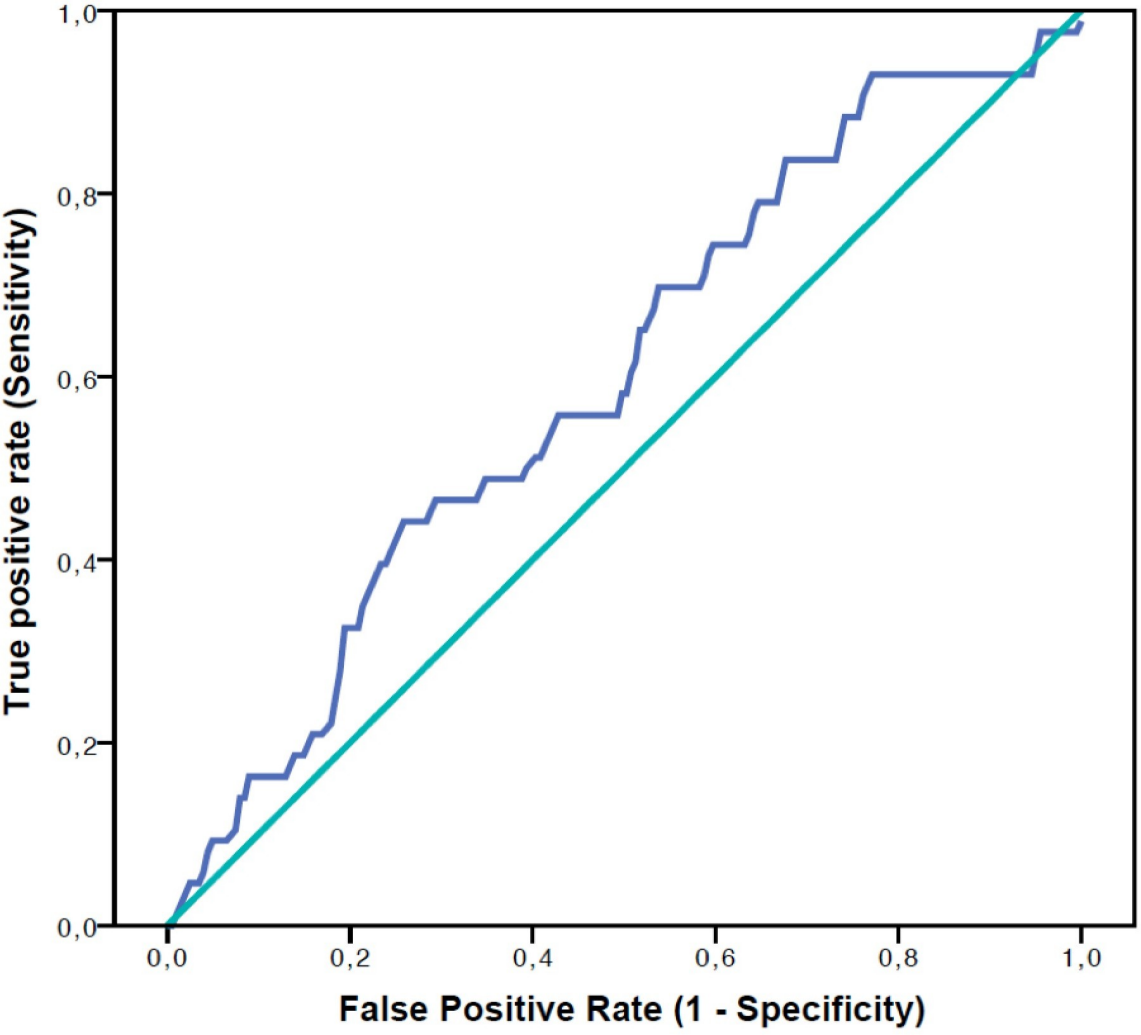
Receiving operative characteristic (ROC) curve for the prediction of live birth based on early luteal progesterone levels during IVF. Area under ROC curve = 0.599 (0.507–0.691), p = 0.042.

The ROC curve for prediction of live birth based on early luteal P_4_ levels (Fig 1) allowed us to find two optimal thresholds and to identify three P_4_ groups: patients (n = 50) who had early luteal P_4_ < 115 nmol/l had a significantly lower live birth rate (6.0%) than patients (n = 122) with P_4_ levels [115–252 nmol/l] (17.2%) and patients (n = 70) with P4 levels > 252 nmol/l (27.1%; p = 0.011) (Table 2). Similarly, the rates of positive hCG (p = 0.043) and clinical pregnancies (p = 0.009) significantly differed across the three groups. The rates of early pregnancy loss were 5/9 (55.6%), 11/33 (33.3%) and 6/27 (22.2%) respectively for the P_4_ groups < 115 nmol/l, [115–252 nmol/l], > 252 nmol/l. The low number of observations did not allow us to find any significant difference across groups for this reproductive outcome (p = 0.182).

### Multiple regression analysis

Due to missing data, the multiple regression analysis using the maternal age, the maternal BMI, the number of follicles > 14 mm and the late follicular P_4_ levels > 4.77 nmol/l was only conducted on 231 patients. The results of this analysis are presented in Table 3. Patients who had early luteal P4 levels < 115 nmol/l had the poorest chance of giving live birth (adjusted OR = 0.10 [0.02–0.52]) compared with patients with early luteal P_4_ > 252 nmol/l. Patients with early luteal P_4_ [115–252 nmol/l] also had a significantly lower chance of giving live birth than patients with P_4_ > 252 nmol/l (adjusted OR = 0.40 [0.18–0.91]). The same significative differences were obtained for the positive hCG and the clinical pregnancies but not for the early pregnancy losses (Table 3).

**Table 3:** Reproductive outcomes in the different luteal progesterone groups.

	OR for positive hCG				OR for clinical pregnancy				OR for live birth				OR for early pregnancy loss			
	N	Crude OR [95% CI]	N	Adjusted OR [95% CI]	N	Crude OR [95% CI]	N	Adjusted OR [95% CI]	N	Crude OR [95% CI]	N	Adjusted OR [95% CI]	N	Crude OR [95% CI]	N	Adjusted OR [95% CI]
N	69/242	242*	63/231	231**	47/242	242*	43/231	231**	43/242	242*	39/231	231**	22/69	69	20/65	65
P4 < 115 nmol/l	9/50	0.35 [0.15–0.83]	8/48	0.26 [0.09–0.76]	4/50	0.20 [0.06–0.64]	3/48	0.13 [0.03–0.53]	3/50	0.17 [0.05–0.617]	2/48	0.10 [0.01–0.52]	5/9	4.37 [0.89–21.61]	5/9	10.42 [0.84–129.16]
P4 115–252 nmol/l	33/122	0.59 [0.32–1.10]	30/117	0.47 [0.24–0.99]	22/122	0.51 [0.26–1.02]	20/117	0.36 [0.16–0.80]	21/122	0.56 [0.27–1.13]	19/117	0.40 [0.18–0.91]	11/33	1.750 [0.55–5.59]	10/31	3.64 [0.72–18.23]
P4 > 252 nmol/l	27/70	1	25/66	1	21/70	1	20/66	1	19/70	1	18/66	1	6/27	1	5/25	1

*In the crude OR estimates, all 242 patients with embryo transfer are included.

**Due to missing data for the covariate late follicular phase in 7 patients, the final follicle count > 14 mm on trigger day for 2 patients, and the BMI for 2 patients, the final adjusted regression model included 231 patients.

## Discussion

To our knowledge, our study is the first to study the effect of the early luteal phase during IVF with the use of oral dydrogesterone for the LPS. Lotus I, a large double-blinded RCT recently demonstrated the noninferiority of oral dydrogesterone compared with micronized vaginal progesterone for LPS in terms of pregnancy rates and tolerability [13]. Several other prospective studies have shown the effectiveness and safety of oral dydrogesterone for LPS during IVF or ICSI cycles and higher satisfaction rates for women compared with micronized vaginal progesterone [7,17–21]. Thus, according to some authors, dydrogesterone may become the new standard for LPS during fresh ET IVF cycles [15].

**Fig 2:**
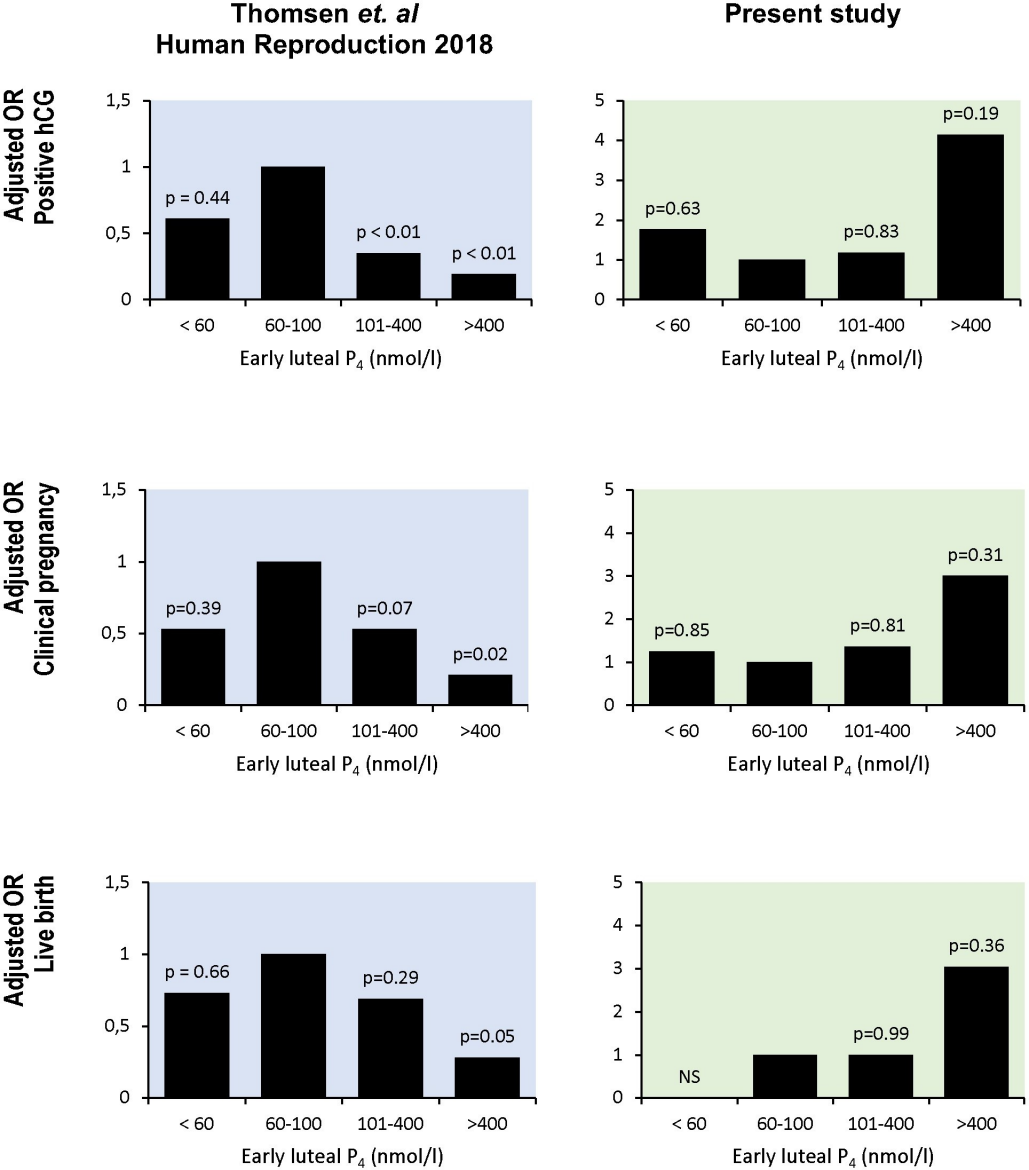
Comparison of the reproductive outcomes of our retrospective cohort (in green, right section, 179 patients) with the Thomsen *et al*. prospective cohort (in blue, left section, 389 patients).

Our results suggest a positive association between early luteal P_4_ levels and reproductive outcomes in IVF treatment with oral dydrogesterone for the luteal support. Therefore, these results are not only inconsistent with the conclusion of Thomsen *et al*. but the complete opposite [11]. After applying the same exclusion criteria as in the Thomsen *et al*. study (age < 41, BMI < 35, and excluding the flare up protocols), only 179 patients were divided into the same P_4_ groups <60 nmol/l; [60–100 nmol/l]; [100–400 nmol/l]; and > 400 nmol/l (respectively n = 5; n = 12; n = 154; n = 8). Our results are compared to those of the Thomsen et al. study in Fig 2. Although the low number of subjects in three of the four groups in this analysis did not allow us to find any significant differences among groups, the results of the two cohorts of patients are clearly inconsistent. The P_4_ group [60–100 nmol/l] that was associated with the best clinical pregnancy rate in the Thomsen *et al*. study had the poorest chance in our study. Similarly, the P_4_ group > 400 nmol/l that was associated with the poorest live birth rate in the aforementioned study was associated with the best live birth rate in our study.

The most plausible hypothesis for these discrepancies is that dydrogesterone interferes with the progesterone secretion by the *corpus lutei*. Indeed, we measured P_4_ levels only and do not report the dydrogesterone levels, which would have required the use of an instrumental chromatographic method [16,22]. Although the influence of dydrogesterone on the secretion of progesterone during the luteal phase has not been fully elucidated, its administration lowers the P_4_ levels [23]. Two studies have also shown the efficacy of dydrogesterone administration to prevent premature LH surges in the context of frozen-thawed ET, suggesting negative feedback on the pituitary gland [23,24]. Although it is clearly impossible with the present study to estimate the respective weights of influence of exogenous dydrogesterone and endogenous progesterone on the outcomes of IVF treatment, our study clearly illustrates that the thresholds suggested by Thomsen et al. are not transposable when dydrogesterone is used for the LPS.

The present study has several limitations. The small sample size forced us to conduct the statistical analysis on early luteal P_4_ cut-off values that were determined in a data-dependent way. This method of analysis is known to bear the risk of finding an effect in the sample while no real difference exists in the population [12,25]. Although we noticed the same trends when the data were analyzed in an objective way–i.e. percentile groups–due to sample size, we were unable to show significant differences using this analysis. Comparability of our P_4_ groups could also be criticized, as there are significant differences between groups. To overcome this issue, we chose to use the exact same variables as Thomsen *et al*. for the multiple regression analysis [11]. They used a Directed Acyclic Graph to identify a minimum set of covariates to adjust for in the statistical analysis. However, in the present study, the sample size was too small to show significant differences between groups by using a classical selection process for the multiple regression. Therefore, we cannot formally exclude a confusion bias. Overall, in addition to the retrospective nature of the study, the lack of statistical power does not allow us to present an irreproachable conclusion and further larger prospective cohort studies must be conducted. Another obvious limitation of the present study is the absence of early luteal phase dydrogesterone level measurements. This missing information prevents us from fully concluding on the pathophysiological mechanisms involved in the relationship between P_4_ levels and reproductive outcomes. Nevertheless, we successfully identified early luteal P_4_ thresholds that–if confirmed by prospective studies–may be used in clinical practice. Indeed, we determined that 21% of our patients who had P_4_ levels below 115 nmol/l had a dramatically poor live-birth rate (6.0%). If confirmed, this finding would encourage physicians to cancel the ET for these patients. The effect of increasing the supplementation is more intangible because the dydrogesterone might induce negative feedback over the progesterone secretion of the *corpus lutei* [23]. Furthermore, increasing progesterone or dydrogesterone supplementation does not proportionally increase the serum levels, and uterine progesterone levels do not correlate well with serum levels [26–29]. These pharmacokinetic data add to the uncertainty regarding strategies to increase the LPS in the case of low early luteal P_4_ levels.

Thus, our results should be interpreted with utmost precautions, and we only conclude that early luteal P_4_ levels and the reproductive outcomes in IVF with oral dydrogesterone for luteal support appear to be positively associated. The current state of knowledge on early luteal P_4_ monitoring is still too weak to allow for consideration of interventional studies or clinical applications. Further larger prospective cohort studies must be conducted to determine reliable thresholds that could be used to personalize LPS.
